# Metabolic engineering of *Saccharomyces cerevisiae* for efficient utilization of pectin-rich biomass

**DOI:** 10.71150/jm.2503001

**Published:** 2025-07-31

**Authors:** Dahye Lee, Fransheska Semidey, Luping Xu, Eun Joong Oh

**Affiliations:** 1 Department of Food Science, Purdue University, West Lafayette, IN 47907, USA; 2 Whistler Center for Carbohydrate Research, Purdue University, West Lafayette, IN 47907, USA

**Keywords:** pectin-rich biomass, *Saccharomyces cerevisiae*, glucose repression, metabolic engineering

## Abstract

Pectin-rich biomass, derived from fruit and citrus processing waste, presents a promising yet underutilized resource for sustainable biofuel and biochemical production. Its low lignin content and high concentrations of fermentable sugars, including D-galacturonic acid, L-arabinose, and D-xylose, make it an attractive feedstock. Unlike lignocellulosic biomass, pectin-rich hydrolysates require milder pretreatment, improving sugar recovery efficiency. However, industrial strains such as *Saccharomyces cerevisiae* exhibit strong glucose preference, limiting the efficient co-fermentation of mixed sugars. While prior reviews have broadly addressed lignocellulosic biomass utilization, this mini-review uniquely centers on the specific metabolic challenges and opportunities associated with pectin-rich feedstocks. In addition to incorporating established strategies for the co-utilization of cellobiose and xylose, we highlight recent advances that allow *S. cerevisiae* to metabolize carbon sources specifically from pectin-rich biomass, such as L-arabinose and D-galacturonic acid—monomers not prevalent in traditional lignocellulosic biomass. By integrating discussions on sugar transport engineering, redox balancing, and pathway optimization, this review offers a comprehensive framework to overcome glucose repression and support efficient co-fermentation of carbon sources from conventional and pectin-rich biomass. Drawing on these advances, we outline practical strategies to enhance fermentation performance and expand the valorization of food processing residues in biomanufacturing.

## Introduction

Food waste is a significant global challenge, with an estimated 30–40% of the food supply lost in the United States alone. In 2014, over 38 million tons of food waste were generated, with only 5% diverted from landfills and incinerators for composting. Reducing food waste by just 15% could feed over 25 million Americans annually (Gunders and Bloom, 2017). The United Nations Environment Programme (UNEP), in its Food Waste Index Report 2024, underscores the persistent issue of household, retail, and food service waste, reinforcing the urgency of mitigation strategies (UNEP, 2024). Food waste valorization presents an opportunity to enhance food sustainability while addressing environmental and economic concerns. Utilizing food waste for biofuel production enables the recovery of energy from underutilized resources that contribute to greenhouse gas emissions. Recent research has demonstrated that food waste is a viable source of minerals and nutrients beneficial for agricultural production. In the short term, this enhances product quality, while in the long term, it contributes to mitigating food insecurity (O'Connor et al., 2021). Furthermore, bioenergy and biocompounds derived from food waste offer innovative possibilities for reimagining waste utilization for human benefit (Lynd et al., 2008; Rabha et al., 2023).

**Fig. 1.**
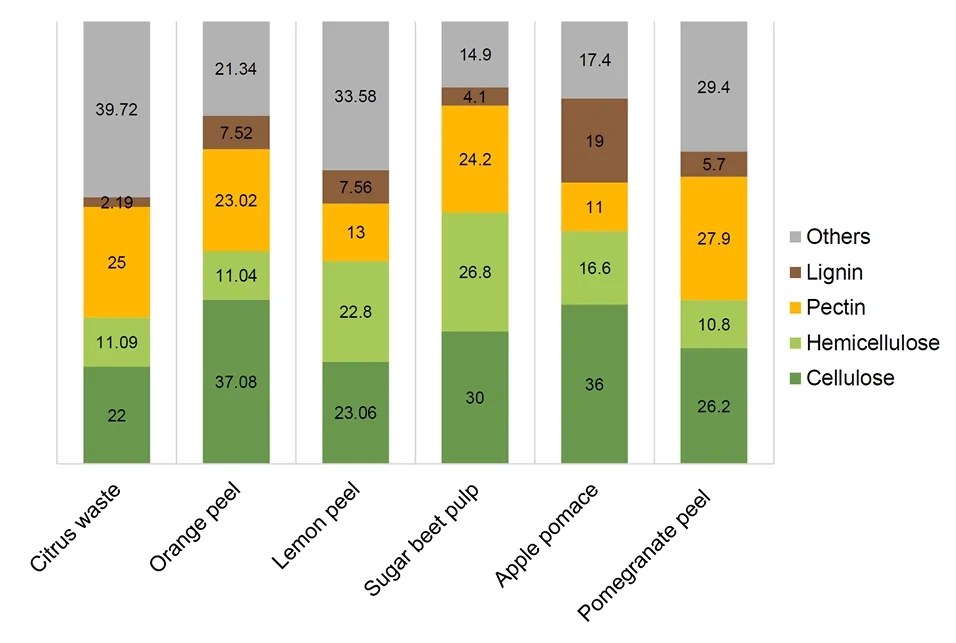
Dry-weight composition of pectin-rich residues. Pectin-rich biomass includes citrus waste (Pourbafrani et al., 2010), orange peel, lemon peel (Taghizadeh-Alisaraei et al., 2017), sugar beet pulp (Zieminski and Kowalska-Wentel, 2017), apple pomace (Pathania et al., 2018), and pomegranate peels (Pocan et al., 2018).

Pectin-rich biomass such as apple pomace, citrus peel, and sugar beet pulp is an underutilized but highly promising resource for biomanufacturing. In contrast to widely studied lignocellulosic biomass, pectin-rich residues offer distinct advantages in both processing efficiency and product versatility, making them an economically and environmentally attractive alternative for industrial applications. Unlike lignocellulosic biomass, which contains a high amount of lignin, pectin-rich biomass is more accessible to enzymatic hydrolysis and requires less intensive pretreatment. This lowers both processing costs and energy input, making pectin-rich residues a more sustainable option for industrial applications (Edwards and Doran-Peterson, 2012). In the citrus industry, residual pulp waste constitutes 55–65% of total fruit mass and contains active compounds such as pectin and simple sugars (Zannini et al., 2021) (Fig. 1). These residues can be repurposed as secondary raw materials, contributing to sustainability efforts. Pectin, a major component of citrus peels, comprises 60–75% D-galacturonic acid among its total sugars and contains other sugar monomers, including L-arabinose, D-galactose, L-rhamnose, and D-xylose (Table 1) (Mohnen, 2008; Yapo et al., 2007). These sugars are valuable substrates for microbial fermentation, particularly when producing bio-based chemicals from food waste. Beyond its role as a carbon source, pectin itself offers significant commercial potential as a renewable biopolymer. It has long been used as a gelling agent, thickener, and stabilizer in food and pharmaceutical products. More recently, interest has grown in its use for developing biodegradable films, foams, and plastic alternatives (Jansen et al., 2024). These functional properties make pectin an attractive material for sustainable product development in both consumer and industrial markets.

Recent advances in metabolic engineering have enabled the development of yeast strains capable of utilizing non-fermentable sugars derived from pectin-rich biomass (Gao et al., 2022; Yang et al., 2020). Optimizing fermentation processes to accommodate mixed sugars in pectin-rich hydrolysates is crucial for improving biomass utilization efficiency. Among industrially relevant yeasts, *Saccharomyces cerevisiae* remains the preferred host for large-scale biomanufacturing due to its robustness, well-characterized genome, and availability of synthetic biology tools (Chen et al., 2018). However, when fermenting sugar mixtures, *S. cerevisiae* prioritizes glucose metabolism, leading to sequential fermentation and reduced volumetric productivity of ethanol and other bioproducts (Trumbly, 1992). The repression of non-glucose sugar metabolism results in residual sugar accumulation, which poses a significant barrier to the economic feasibility of biomass hydrolysate fermentation. Metabolic engineering strategies have thus focused not only on improving single-sugar fermentations but also on overcoming glucose repression in *S. cerevisiae* to enhance biochemical production. This review explores research on the effective utilization of sugars derived from pectin-rich biomass, emphasizing strategies for optimizing *S. cerevisiae* metabolism to enhance bioproduct yields.

## Metabolic Pathways of Non-glucose Carbon Sources from Pectin-rich Biomass

### D-Galacturonic acid

The backbone of pectin consists primarily of α-1,4-linked D-galacturonic acid, which *S. cerevisiae* is unable to naturally ferment. Moreover, because D-galacturonic acid is more oxidized than neutral hexose and pentose sugars, its fermentation requires additional NADPH cofactors for ethanol production (Richard and Hilditch, 2009). With a dissociation constant (*pK_a_*) of 3.51, D-galacturonic acid exists in both dissociated and undissociated forms at fermentation-relevant pH levels. Previous studies have shown that, in its undissociated form, D-galacturonic acid significantly inhibits sugar fermentation, reducing galactose consumption by 87% and severely impairing D-xylose and L-arabinose utilization in engineered yeast strains (Huisjes et al., 2012a). These inhibitory effects, coupled with the metabolic limitations of *S. cerevisiae*, present a major challenge in the efficient utilization of pectin-rich biomass.

**Fig. 2.**
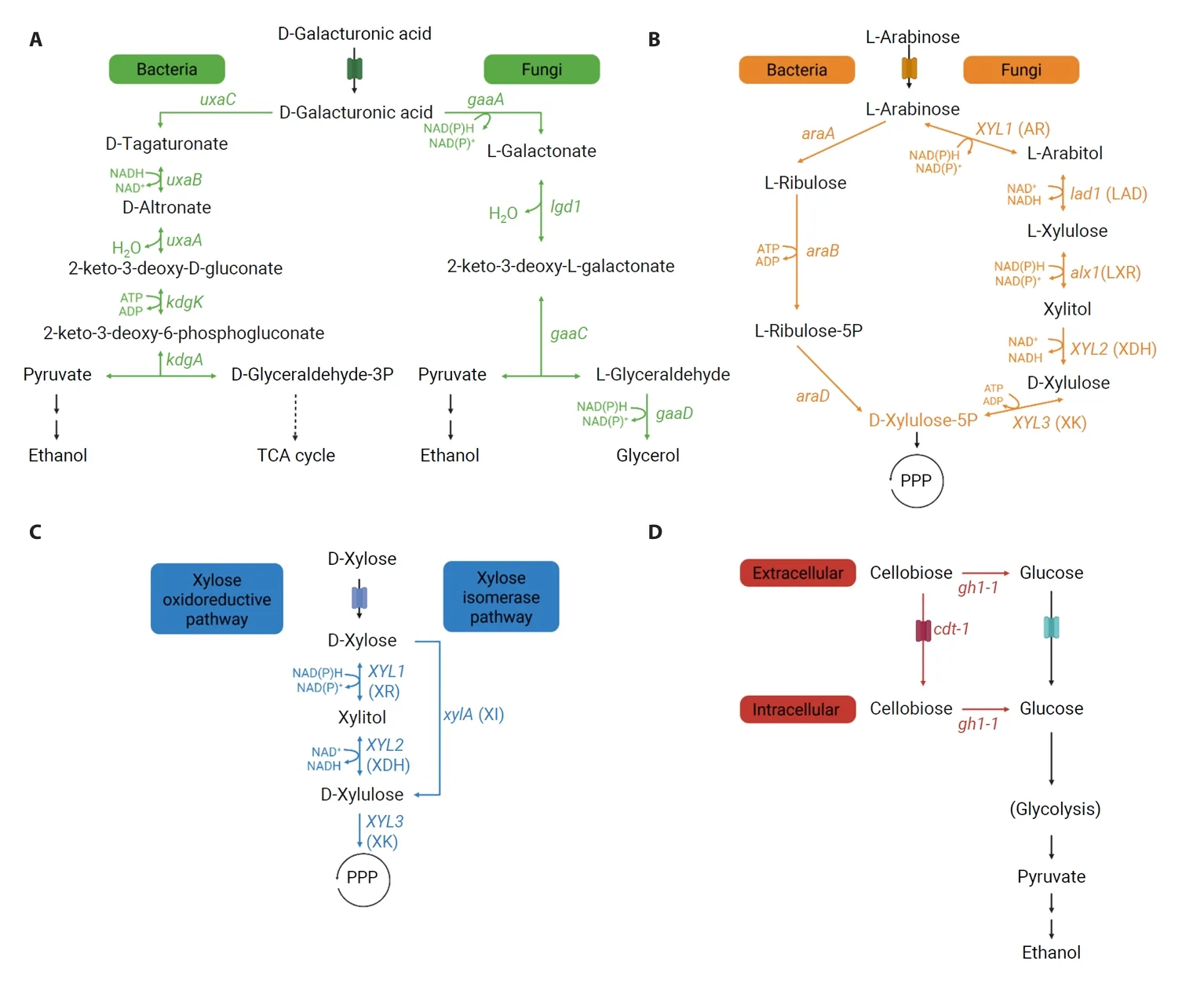
Metabolic pathways of D-galacturonic acid, L-arabinose, D-xylose, and cellobiose in fungi and bacteria. (A) *uxaC*, uronate isomerase; *uxaB*, tagaturonate reductase; *uxaA*, altronate dehydratase; *kdgK*, 2-keto-3-deoxy-gluconate kinase; *kdgA*, 2-keto-3-deoxy-phosphogluconate aldolase; *gaaA*, D-galacturonic acid reductase; *lgd1*, L-galactonate dehydratase; *gaaC*, 2-keto-3-deoxy-L-galactonate aldolase; *gaaD*, glyceraldehyde reductase. (B) *XYL1* (AR), aldose reductase; *lad1* (LAD), L-arabitol-4-dehydrogenase; *alx1* (LXR), L-xylulose reductase; *XYL2* (XDH), xylitol dehydrogenase; *XYL3* (XK), xylulokinase; *araA*, L-arabinose isomerase; *araB*, L-ribulokinase; *araD*, L-ribulose-5-phosphate 4-epimerase. (C) *xylA* (XI), xylose isomerase. (D) *cdt-1*, cellodextrin transporter; *gh1-1*, β-glucosidase. Heterologous pathways for each metabolism are highlighted with distinct colors.

Several studies have attempted to introduce heterologous pathways to enable efficient metabolism of D-galacturonic acid from pectin-rich hydrolysates. D-Galacturonic acid can be metabolized by bacteria such as *Escherichia coli*, *Agrobacterium tumefaciens* (Richard and Hilditch, 2009), *Lactococcus lactis* (Huisjes et al., 2012b), and *Lactobacillus suebicus* (Valk et al., 2019), as well as by fungi including *Hypocrea jecorina*, *Aspergillus niger* (Richard and Hilditch, 2009), *Trichoderma reesei* (Biz et al., 2016), and *Rhodosporidium toruloides* (Protzko et al., 2019). In bacteria, the enzymes uronic acid isomerase and mannonate dehydratase convert D-galacturonic acid into 2-keto-3-deoxy-D-gluconate (KDG), a key intermediate in bacterial D-galacturonic acid metabolism. To introduce this pathway into *S. cerevisiae*, *uxaC* (uronic acid isomerase) and *uxaB* (mannonate dehydratase) from *E. coli* were heterologously expressed. Although this approach enabled partial conversion of D-galacturonic acid, the engineered strains showed low growth rates and poor substrate utilization (Huisjes et al., 2012b). In fungal metabolism, D-galacturonic acid is initially reduced to L-galactonate by D-galacturonic acid reductase via the predominant reductive pathway. The reductases from *T. reesei* and *A. niger* have been extensively studied. The enzyme *TrGar1* from *T. reesei* primarily utilizes NADPH as a cofactor (Kuorelahti et al., 2005), while *AnGaaA* from *A. niger* preferentially uses NADPH but can utilize NADH to a lesser extent (Martens-Uzunova and Schaap, 2008). To enhance D-galacturonic acid metabolism in *S. cerevisiae*, heterologous expression of key genes has been investigated. The genes *gaaA*, *gaaC*, and *gaaD* from *A. niger* encoding D-galacturonic acid reductase, 2-keto-3-deoxy-L-galactonate aldolase, and glyceraldehyde reductase together with *lgd1* from *T. reesei* encoding D-galactonate dehydratase were successfully expressed in *S. cerevisiae* for ethanol production from D-galacturonic acid (Biz et al., 2016) (Fig. 2A).

### L-Arabinose

L-Arabinose utilization has been studied significantly less than xylose utilization, despite being the third most abundant sugar in lignocellulosic biomass after glucose and xylose. However, pectin-rich alternative feedstock has significantly higher concentrations of L-arabinose (Xiao and Anderson, 2013). As one of the predominant pentoses in pectin-derived oligosaccharides, L-arabinose holds great potential as a carbon source for biotechnological applications. Given its abundance in underutilized pectin-rich biomass, further research is necessary to optimize its metabolic integration and industrial utilization (Kumar et al., 2024).

Various metabolic engineering strategies have been employed to enable efficient L-arabinose fermentation in *S. cerevisiae*. The *araA* gene encoding L-arabinose isomerase from *Bacillus subtilis* (Becker and Boles, 2003; Wiedemann and Boles, 2008), *B. licheniformis* (Wang et al., 2019; Wiedemann and Boles, 2008), and *Lactobacillus plantarum* (Wang et al., 2013), along with the *araB* (ribulokinase) and *araD* (L-ribulose-5-phosphate 4-epimerase) genes from *E. coli* (Wang et al., 2019; Wiedemann and Boles, 2008) or *L. plantarum* (Wang et al., 2017b), has been introduced into engineered *S. cerevisiae* strains to support anaerobic L-arabinose fermentation. Ethanol production from these strains ranged from 6 to 9 g/L utilizing 20 g/L of L-arabinose. In L-arabinose-fermenting fungi, L-arabinose is converted to D-xylulose through a series of reduction and oxidation reactions catalyzed by aldose reductase (AR), L-arabitol-4-dehydrogenase (LAD), L-xylulose reductase (LXR), and xylitol dehydrogenase (XDH) (Bettiga et al., 2009). To engineer xylose-fermenting *S. cerevisiae* for L-arabinose utilization, heterologous AR, XDH, and XK from *Scheffersomyces stipitis* are introduced, followed by the expression of *T. reesei* *lad1* and *Ambrosiozyma monospora* *alx1* for LAD and LXR activity (Bera et al., 2010; Bettiga et al., 2009; Metz et al., 2013). D-xylulose is then phosphorylated into D-xylulose-5-phosphate by D-xylulokinase (XK). Both bacterial and fungal pathways converge at D-xylulose-5-phosphate, linking L-arabinose metabolism to the pentose phosphate pathway (PPP) (Bettiga et al., 2009; Verho et al., 2004) (Fig. 2B).

The cofactor preferences of AR and LXR vary among fungal species. The initial reductase enzyme typically favors NADPH, whereas the two dehydrogenases strictly require NAD^+^ (Fonseca et al., 2007). As a result, the redox balance necessary for efficient cell growth is maintained under aerobic conditions. However, under oxygen-limited environments, NAD^+^ availability becomes restricted, leading to L-arabitol accumulation, impaired L-arabinose metabolism, and reduced ethanol production (Richard et al., 2003).

### D-Xylose

Xylose catabolism occurs via three distinct pathways in microorganisms, but only two have been introduced into *S. cerevisiae*. In the first pathway, xylose is reduced to xylitol by xylose reductase (XR), encoded by *XYL1* (Verduyn et al., 1985). Xylitol is then oxidized to D-xylulose by xylitol dehydrogenase (XDH), encoded by *XYL2* (Rizzi et al., 1989). D-Xylulose is subsequently phosphorylated by xylulokinase (XK), enabling its entry into central carbon metabolism. Some bacteria and fungi metabolize xylose via xylose isomerase (XI) pathway, in which xylose is directly converted to D-xylulose by xylose isomerase (Kuyper et al., 2005). In both pathways, xylulose-5-phosphate enters the PPP and glycolysis, driving energy production and biosynthesis (Fig. 2C).

Various sources of XR and XDH have been introduced into *S. cerevisiae*, with the most common enzymes derived from *S. stipitis*. Compared to the XI pathway, the XR-XDH pathway is thermodynamically favorable and enables faster xylose consumption and ethanol production (Karhumaa et al., 2005, 2007). However, XR preferentially utilizes NADPH rather than NADH as a cofactor, whereas XDH exclusively relies on NAD^+^ (Hahn-Hägerdal et al., 2007). This cofactor mismatch disrupts intracellular redox balance, leading to xylitol and glycerol accumulation during xylose fermentation, which reduces carbon assimilation and ethanol yield. To address these challenges, extensive efforts have been directed to optimizing intracellular redox balance and engineering XR and XDH variants with altered cofactor specificities to enhance metabolic efficiency (Jeppsson et al., 2006; Krahulec et al., 2009; Lee et al., 2012; Runquist et al., 2010).

## Microbial Strategies for Cellulose utilization

Cellodextrins are glucose polymers of varying lengths produced from the enzymatic breakdown of cellulose. As a linear polymer of glucose, cellulose provides structural support to plant cell walls and constitutes a major component of agro-industrial and food waste (Ahmad Khorairi et al., 2023; Garnett et al., 2024). Research has increasingly focused on extracting and utilizing cellulose waste for biofilm production, microbial protein synthesis, and organic acid generation, thereby contributing to a sustainable bioeconomy (Cakmak and Dekker, 2022; Das and Singh, 2004). The microbial conversion of cellulose into biofuels and bio-based chemicals offers an attractive method for harnessing this abundant carbon source.

Since wild-type *S. cerevisiae* lacks the native ability to metabolize cellodextrins or cellulose, significant efforts have been made to engineer heterologous pathways for cellulose utilization. Two primary strategies have been developed: (1) extracellular hydrolysis of cellulose into glucose, which is subsequently transported into the cell, and (2) partial breakdown of cellulose into shorter oligomers such as cellobiose, which are imported intracellularly for further degradation (Fig. 2D).

In cellulolytic microorganisms, cellulase enzymes hydrolyze the β-1,4 linkages in cellulose. Many cellulases possess a carbohydrate-binding module (CBM) that enhances substrate binding, facilitating prolonged interaction with insoluable cellulose (McDonald et al., 2012). Aerobic microorganisms secrete individual cellulases that act synergistically, whereas anaerobic microorganisms employ cellulosomes, multi-enzyme complexes that anchor to cellulose and enhance degradation efficiency (Kuhad et al., 2016). The scaffolding protein in cellulosomes binds to dockerin domains on catalytic enzymes via dockerin-cohesin interactions, securing the complex to cellulose (Vera et al., 2021). Cellulases function through distinct catalytic mechanisms. Endoglucanases (EC 3.2.1.4) cleave cellulose randomly at amorphous regions, generating new chain ends. Exoglucanases (cellobiohydrolases, EC 3.2.1.91) hydrolyze cellulose from these chain ends, targeting crystalline regions and releasing cellobiose as the main product. β-Glucosidases (EC 3.2.1.21) complete cellulose hydrolysis by converting cellobiose into glucose monomers (Wilson, 2011).

Due to the diversity of cellulases, selecting optimal enzymes for heterologous expression in *S. cerevisiae* remains a key challenge. Both bacterial and fungal cellulases have been investigated. Cellulose-degrading bacteria inhabit diverse environments, including *Actinobacteria*, *Firmicutes*, *Proteobacteria*, and *Bacteroidetes*, which contribute to complex carbohydrate degradation, such as in human gut microbiota (Flint et al., 2012; Li et al., 2021; Rabha et al., 2023). To engineer *S. cerevisiae* for cellulose degradation, researchers have successfully introduced bacterial cellulases. Endoglucanase, cellobiohydrolase, and β-glucosidase from *Ruminiclostridium cellulolyticum* and *Acetivibrio thermocellus* have been expressed in *S. cerevisiae*, enabling ethanol production from cellulose (Tsai et al., 2013). Given the complexity of cellulosomes, alternative systems have focused on fungal cellulases. Enzymes from *T. reesei*, including endoglucanase and exoglucanase, combined with *A. aculeatus* β‐glucosidase, have been expressed in *S. cerevisiae* to convert cellulose into glucose for ethanol fermentation (Inokuma et al., 2014; Liu et al., 2017b; Matano et al., 2012; Wen et al., 2010).

## Engineering Sugar Transporters for Enhanced Fermentation in *S. cerevisiae*

Efficient sugar uptake is a critical factor in fermentation performance, particularly when utilizing pectin-rich biomass. However, the native sugar transporters in *S. cerevisiae* often exhibit low affinity for non-native sugars, limiting metabolic efficiency. To overcome this limitation, recent advances in transporter engineering have focused on improving the uptake of L-arabinose, D-galacturonic acid, and cellodextrins. These efforts aim to enhance carbon utilization and overall process efficiency.

### D-Galacturonic acid transporter

Microorganisms have evolved specialized transporter systems to facilitate the uptake of pectin-derived degradation products, particularly D-galacturonic acid. Several transporters have been identified in *Neurospora crassa*, *A. niger*, and *T. reesei* (Benz et al., 2014; Protzko et al., 2018; Wagner et al., 2021). The *N. crassa* GAT-1 transporter has been shown to mediate rapid D-galacturonic acid uptake, highlighting its potential utility in *S. cerevisiae* (Benz et al., 2014). Additionally, the introduction of the *A. niger* GatA transporter, combined with pH optimization, has been shown to enhance D-galacturonic acid consumption in engineered *S. cerevisiae*, enabling the co-utilization of D-galacturonic acid and glucose (Protzko et al., 2018).

Despite these advances, uptake and conversion efficiency remain suboptimal. In *S. cerevisiae*, D-galacturonic acid uptake is mediated by hexose transporters, and its consumption is inhibited by the presence of D-glucose (Protzko et al., 2018). This glucose-mediated inhibition represents a major challenge for the efficient co-utilization of mixed sugars in pectin-rich hydrolysates. Although heterologous expression of the *A. niger* GatA transporter improves D-galacturonic acid uptake, it does not fully alleviate glucose repression. These findings emphasize the need for further metabolic engineering to optimize D-galacturonic acid transport and metabolism in *S. cerevisiae*. Strategies to relieve glucose repression and improve co-consumption of D-galacturonic acid and glucose are essential for industrial applications.

### L-Arabinose transporter

L-Arabinose uptake represents a major bottleneck in its metabolism by *S. cerevisiae*. The native galactose permease, Gal2, transports L-arabinose with low efficiency (Becker and Boles, 2003; Oehling et al., 2018). To enhance L-arabinose transport, modifications to Gal2 have been explored based on molecular modeling of its substrate-binding site. The F85S mutation in Gal2p enhanced L-arabinose transport efficiency and weakened the transporter’s preference for glucose, leading to significantly improved growth rates of *S. cerevisiae* on L-arabinose as the sole carbon source (Wang et al., 2017a). Beyond modifications to native transporters, several heterologous L-arabinose transporters have been identified and expressed in *S. cerevisiae*. Transporters from *N. crassa*, *Myceliophthora thermophila* (Li et al., 2015), *Penicillium chrysogenum* (Bracher et al., 2018), *Arabidopsis thaliana*, *S. stipitis* (Subtil and Boles, 2011), and *T. reesei* (Havukainen et al., 2021) have been investigated for their potential to enhance L-arabinose uptake. Among these, *N. crassa* Lat-1 has been reported as an efficient L-arabinose transporter, exhibiting an uptake rate of 116.7 mmol/h/g dry cell weight—two orders of magnitude higher than that of *S. cerevisiae* Gal2 (0.13 mmol/h/g dry cell weight) (Li et al., 2015). Additionally, *P. chrysogenum* AraT has been characterized as a high-affinity L-arabinose transporter but is inhibited by glucose and xylose (Bracher et al., 2018; Verhoeven et al., 2018). A recent study identified *T. reesei* Trire2_104072 as a highly specific L-arabinose transporter with superior performance compared to Lat-1. This transporter facilitates high-affinity L-arabinose uptake with minimal inhibition by glucose and xylose, making it a promising candidate for metabolic engineering in pentose-utilizing yeast strains (Havukainen et al., 2021).

Despite the successful introduction of heterologous transporters, substantial improvements in L-arabinose fermentation and its simultaneous utilization with glucose have not yet been achieved. This suggests that the primary limitation in L-arabinose metabolism in engineered *S. cerevisiae* is an imbalance of intracellular enzymatic activities involved in the L-arabinose metabolic pathway. A reduction in L-ribulokinase activity, combined with enhanced transaldolase activity, has been identified as a key factor in improving L-arabinose utilization in *S. cerevisiae* (Becker and Boles, 2003).

### Cellodextrin transporter

Cellulose hydrolysis using exogenous cellulase cocktails is often hindered by weak β-glucosidase activity, which limits the efficient breakdown of cellobiose into glucose. Previous studies have demonstrated that the low catalytic efficiency and substrate inhibition of fungal β-glucosidases significantly restrict cellobiose conversion, reducing overall hydrolysis efficiency (Chauve et al., 2010). Additionally, high glucose concentrations interfere with the simultaneous utilization of other sugars such as xylose and arabinose and induce feedback inhibition, which suppresses cellulase activity and impairs cellulose degradation (Lynd et al., 2002). To address these challenges, intracellular cellobiose hydrolysis has been explored as an alternative approach to minimize extracellular glucose accumulation and alleviate glucose-induced inhibition. Redirecting cellobiose hydrolysis into the intracellular space enhances mixed sugar utilization, effectively overcoming glucose repression and improving hydrolysis efficiency in lignocellulose-based biorefineries (Parisutham et al., 2017).

In cellulose-degrading fungi, efficient uptake of cellodextrins and cellobiose is essential for cellulose metabolism. Instead of relying solely on extracellular hydrolysis, some microorganisms transport cellodextrins intracellularly and hydrolyze them using β-glucosidase. These transporters, commonly found in fungi, also act as transceptors that sense environmental cellulose and regulate cellulolytic enzyme expression. For instance, *CDT-1* and *CDT-2* from *N. crassa* facilitate cellodextrin transport in *S. cerevisiae*, while their deletion impairs cellulose sensing and cellulase gene induction (Znameroski et al., 2014). Similar transporters have been identified, including *CRT1* and *STP1* in *T. reesei* (Zhang et al., 2013), *CLTA* and *CLTB* in *A. nidulans* (dos Reis et al., 2016), and *CdtC*, *CdtD*, and *CdtG* in *P. oxalicum* (Li et al., 2013). Recently, *T. reesei* Tr44175 was discovered as a broad-specificity transporter capable of facilitating the uptake of cellobiose, cellotriose, cellotetraose, and sophorose (Nogueira et al., 2024).

Recombinant expression of *N. crassa* cellodextrin transporter and β-glucosidase in *S. cerevisiae* has enabled cellobiose fermentation, but its efficiency remains lower than extracellular β-glucosidase (Ha et al., 2011a; Lee et al., 2013; Oh et al., 2016; Yuan and Zhao, 2013). To optimize transporter function, directed evolution has been employed to introduce targeted mutations that enhance cellobiose uptake and hydrolysis. Error-prone PCR-based mutagenesis of *N. crassa* *CDT-1* and *GH1-1* identified mutations (L173H in *GH1-1* and C82S in *CDT-1*) that increased cellobiose consumption by 49% and ethanol productivity by 64% (Eriksen et al., 2013). To improve transporter performance under industrial conditions, particularly in acidic environments, directed evolution of *N. crassa* *CDT-2* resulted in mutations (I96N and T487A) that enhanced cellobiose uptake by 30% and ethanol yield by 31% (Oh et al., 2017). Further modification, including the introduction of Q207H, N311H, and I505T mutations, increased ethanol titers by 28% under anaerobic conditions (Lian et al., 2014). Additionally, Kim et al. (2018) identified a *CDT-2* variant (N306I) that improved transporter efficiency and accelerated cellobiose fermentation.

An alternative approach involved engineering the *S. stipitis* hexose transporter *HXT2.4* for improved cellobiose uptake in *S. cerevisiae*. Directed evolution identified the A291D mutation, which enhanced fermentation efficiency, while saturation mutagenesis at position 291 further optimized transport activity. This approach allowed engineered yeast to consume 78 g/L of cellobiose in 24 h, compared to 56 g/L over 60 h in the wild-type strain (Ha et al., 2013). These studies highlight the potential of transporter engineering to improve industrial-scale cellobiose fermentation.

## Metabolic Engineering of the Pentose Phosphate Pathway for Enhanced Pentose Utilization in *S. cerevisiae*

Efficient metabolism of pectin-derived sugars, such as D-galacturonic acid, L-arabinose, and D-xylose, in *S. cerevisiae* is constrained by limited carbon flux through the PPP and imbalances in redox cofactors. The PPP plays a central role in converting pentose sugars into glycolysis intermediates while also generating NADPH, a crucial redox cofactor essential for biosynthesis, redox homeostasis, and oxidative stress resistance. However, disruptions in PPP flux and imbalanced cofactor levels can lead to inefficient pentose metabolism, metabolic bottlenecks, and reduced fermentation efficiency (Stincone et al., 2015).

To address these challenges, metabolic engineering efforts have focused on optimizing both the oxidative and non-oxidative branches of the PPP. This section explores strategies to enhance PPP function, regulate NADPH production, and improve pentose sugar assimilation.

### Engineering the oxidative PPP for NADPH regulation

In *S. cerevisiae*, NADH primarily participates in catabolic processes, such as glycolysis and fermentation, while NADPH plays a vital role in anabolic pathways, including fatty acid synthesis, nucleotide biosynthesis, and oxidative stress resistance. The oxidative and reductive reactions of metabolism rely on NADP^+^ and NADPH, which serve as intracellular electron carriers that drive redox reactions. Due to their distinct metabolic roles, the equilibrium between these cofactors varies. Under aerobic, steady-state chemostat conditions with glucose as the sole carbon source, the NAD^+^/NADH ratio in wild-type *S. cerevisiae* was reported to be approximately 100:1 (Canelas et al., 2008). In contrast, the NADP^⁺^/NADPH ratio has been observed to be around 1:100 during respiratory glucose metabolism (Murray et al., 2011). Since pentose metabolism requires a high supply of NADPH, maintaining an optimal balance between NADH and NADPH is crucial for efficient sugar assimilation and fermentation. Disruptions in this balance impair carbon flux, compromise redox homeostasis, and reduce fermentation performance. This underscores the need for targeted metabolic engineering strategies to optimize PPP activity.

The oxidative PPP serves as the primary source of NADPH, supporting biosynthesis, oxidative stress resistance, and pentose sugar metabolism. Efficient assimilation of D-galacturonic acid, L-arabinose, and D-xylose depends on a well-regulated NADPH supply. Enhancing NADPH availability can be achieved by overexpressing of *ZWF1*, which encodes glucose-6-phosphate dehydrogenase, the enzyme that initiates the oxidative PPP. Introducing *ZWF1* under a constitutive promoter via a multi-copy plasmid enhances NADPH availability, improving glucose and xylose utilization (Liu et al., 2017a). A mutant *ZWF1* with increased expression levels and total NADPH production has also been shown to facilitate efficient xylose utilization (Zha et al., 2014). Furthermore, overexpression of *ZWF1* alone increased chelerythrine production by 14.39%, while co-expression with *GND1*, which encodes 6-phosphogluconate dehydrogenase, led to an additional 9.83% improvement by further enhancing NADPH supply (Zhu et al., 2024). These findings highlight the synergistic benefits of enhancing both steps of the oxidative PPP.

### Engineering the non-oxidative PPP for enhanced carbon flux

**Fig. 3.**
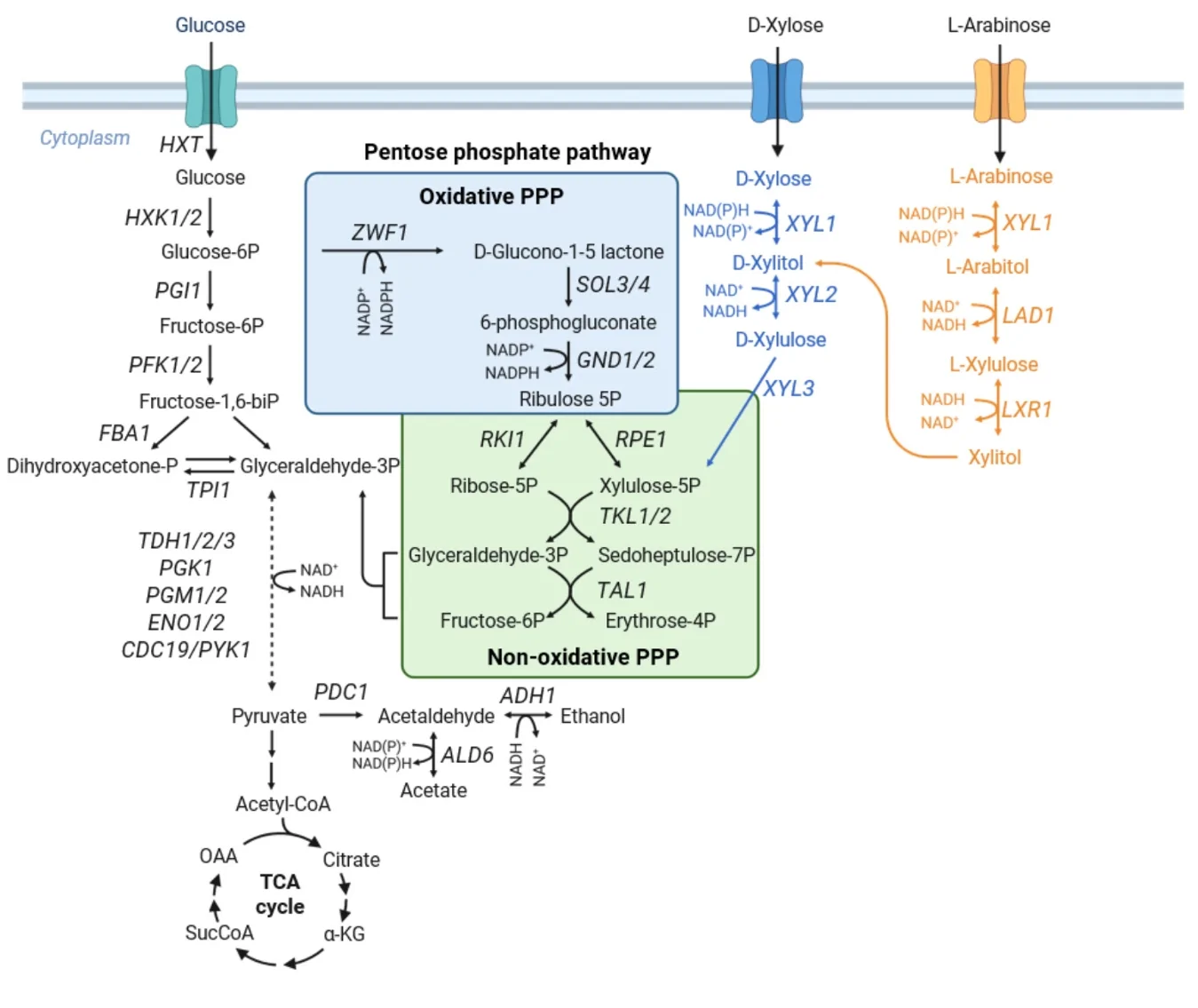
Schematic drawing of central carbon metabolism of *S. cerevisiae*, including glycolysis, the pentose phosphate pathway (PPP), and the tricarboxylic acid (TCA) cycle. Glycolysis: *HXK1/2*, hexokinase; *PGI1*, phosphoglucose isomerase; *PFK1/2*, phosphofructokinase; *FBA1*, fructose-bisphosphate aldolase; *TPI1*, triosephosphate isomerase; *TDH1/2/3*, glyceraldehyde-3-phosphate dehydrogenase; *PGK1*, phosphoglycerate kinase; *PGM1/2*, phosphoglycerate mutase; *ENO1/2*, enolase; *CDC19*/*PYK1*, pyruvate kinase. Fermentation and TCA cycle: *PDC1*, pyruvate decarboxylase; *ADH1*, alcohol dehydrogenase; *ALD6*, aldehyde dehydrogenase; acetyl-CoA; citrate; α-KG, α-ketoglutarate; SucCoA, succinyl-CoA; OAA, oxaloacetate. Oxidative PPP: *ZWF1*, glucose-6-phosphate dehydrogenase; *SOL3/4*, 6-phosphogluconolactonase; *GND1/2*, 6-phosphogluconate dehydrogenase. Non-oxidative PPP: *RKI1*, ribose-5-phosphate isomerase; *RPE1*, ribulose-5-phosphate epimerase; *TAL1*, transaldolase; *TKL1/2*, transketolase. Pentose sugar metabolism: *XYL1*, xylose reductase; *XYL2*, xylitol dehydrogenase; *XYL3*, xylulokinase; *LAD1*, L-arabitol-4-dehydrogenase; *ALX1*, L-xylulose reductase. Heterologous pathways for xylose and arabinose metabolism are highlighted in blue and yellow, respectively.

The non-oxidative branch of the PPP plays a critical role in redirecting pentose sugars into glycolysis. However, bottlenecks in pentose metabolism often arise due to inefficient carbon flux through this pathway, leading to sugar accumulation and limited glycolytic entry. Studies in *S. cerevisiae* strains expressing *XYL1* and *XYL2* have shown that the accumulation of sedoheptulose-7-phosphate, coupled with low levels of fructose-1,6-bisphosphate, restricts carbon flux into glycolysis (Kötter and Ciriacy, 1993). This suggests that optimizing the non-oxidative PPP is essential for improving both xylose and L-arabinose assimilation. Since L-arabinose is converted into xylulose-5-phosphate before entering central metabolism, it follows the same non-oxidative PPP pathway as xylose (Fig. 3). Consequently, similar metabolic engineering strategies that enhance PPP flux are expected to improve L-arabinose utilization in engineered *S. cerevisiae*.

To enhance flux through the non-oxidative PPP, metabolic engineering efforts have focused on overexpressing key enzymes involved in pentose interconversion. Overexpression of *TAL1* (transaldolase) and *TKL1* (transketolase) has been shown to increase pentose flux and significantly improve xylose assimilation rates (Matsushika et al., 2012; Walfridsson et al., 1995). Additionally, overexpression of *XKS1* (xylulokinase), *RKI1* (ribose 5-phosphate isomerase), and *RPE1* (ribulose 5-phosphate epimerase) enhances pentose sugar metabolism by improving interconversion for glycolytic entry (Karhumaa et al., 2005; Kuyper et al., 2005; Qi et al., 2015). Further optimization has involved deleting *PHO13*, a phosphatase that affects pentose phosphate metabolism, to enhance xylose utilization in engineered *S. cerevisiae* strains expressing pentose-assimilating genes (Kim et al., 2015). Additionally, the pentose-fermenting *S. cerevisiae* strain YE9, engineered with a fungal D-galacturonic acid pathway and deletion of *PHO13*, demonstrated efficient co-consumption of D-galacturonic acid and xylose in mixed-sugar media while exhibiting reduced susceptibility to catabolic repression (Jeong et al., 2020). These findings highlight the importance of optimizing the non-oxidative PPP and fine-tuning regulatory mechanisms to alleviate metabolic bottlenecks, allowing for the efficient integration of D-galacturonic acid, L-arabinose, and D-xylose into glycolysis and central carbon metabolism.

## Regulatory Engineering for Improved Non-glucose Carbon Source Fermentation in *S. cerevisiae*

**Fig. 4.**
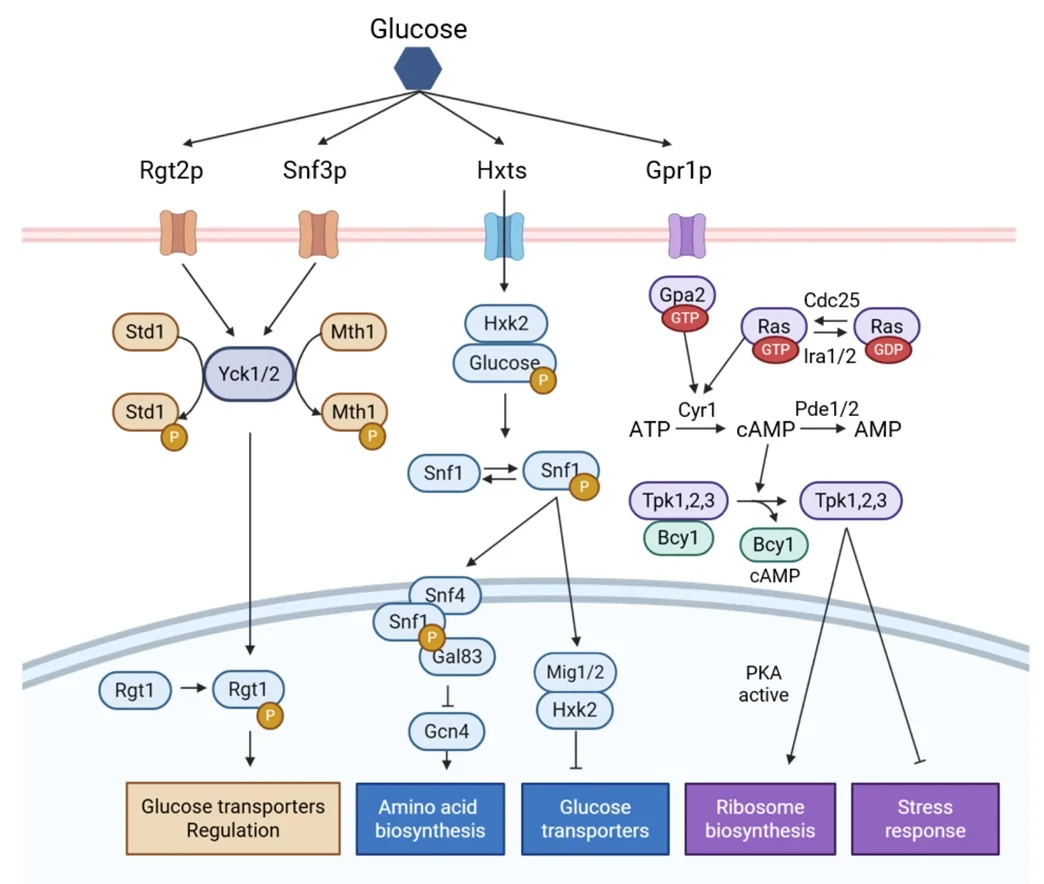
Signal pathways affecting xylose utilization in *S. cerevisiae*. Rgt2p, Snf3p, Gpr1p, and Hxts (Hexose transporters) are cell membrane proteins. Rgt2, Snf3, and Gpr1 act as glucose sensors that signal the Snf3/Rgt2 and cAMP/PKA pathways, respectively. Circles with *P* indicate phosphorylated targets. The Snf3p/Rgt2p pathway (orange) controls the expression of hexose transporters in response to extracellular glucose. The Snf1p/Mig1p pathway (blue) regulates the expression of genes associated with alternative carbon sources in response to intracellular phosphorylated glucose levels. The cAMP/PKA pathway (purple) regulates cellular growth, homeostasis, and stress responses, coordinating metabolic adaptations based on glucose availability.

Glucose repression in *S. cerevisiae* is controlled by multiple mechanisms, including glucose phosphorylation by *SNF1*, repression of alternative sugar metabolism via the cAMP/PKA pathway, and regulation through *RGT2* and *SNF3* (Fig. 4). These regulatory networks strongly influence sugar preference, often resulting in sequential rather than simultaneous sugar consumption. Efficient co-fermentation of carbon sources derived from pectin-rich biomass in *S. cerevisiae* remains constrained by glucose availability. In the presence of glucose, yeast cells prioritize glucose metabolism by upregulating transporters, the PPP, and glycolysis while reducing tricarboxylic acid (TCA) cycle activity (Lee et al., 2021). However, upon glucose depletion, cells shift to an alternative metabolic state, leading to changes in transcriptional regulation and enzyme activities in central carbon flux, which in turn affects the fermentation of D-xylose, L-arabinose, and D-galacturonic acid (Souto-Maior et al., 2009). Therefore, rewiring regulatory networks has been a major focus in metabolic engineering to improve pentose fermentation efficiency.

### Snf3/Rgt2 pathway

Xylose and arabinose uptake in *S. cerevisiae* depends on hexose transporters, whose expression is regulated by the *RGT2*/*SNF3*-*RGT1* regulatory system. Rgt2 and Snf3 are cell membrane proteins that sense extracellular glucose and transmit a signal into the cell. Following glucose detection, the co-inhibitors of Mth1 and Std1 undergo phosphorylation by Yck1/2 and subsequent degradation. This process alleviates Rgt1-mediated inhibition of hexose transporter gene transcription. However, these sensors exhibit limited responsiveness to xylose or arabinose, restricting efficient uptake. Deletion of *RGT1* has been reported to improve xylose utilization, suggesting a potential strategy for enhancing arabinose uptake as well. Additionally, Rgt2 and Snf3 function as poor xylose sensors, indicating that engineering glucose-independent sugar transport regulation could enhance pentose metabolism (Wu et al., 2020).

### Snf1 pathway

The Snf1 protein kinase serves as a central regulator of glucose repression and controls carbon metabolism by phosphorylating transcription factors such as Mig1 and Rgt1. The role of the Snf1 regulatory pathway in xylose metabolism has been extensively investigated. Although the Snf1 pathway does not respond to xylose, its deletion enhances xylose consumption in mixed-sugar environments (Brink et al., 2016; Cai et al., 2018). Disruption of *MIG1*, a transcriptional repressor phosphorylated by Snf1 that regulates hexose transporter genes, has minimal impact on xylose fermentation or glucose-xylose co-fermentation (Roca et al., 2004). However, simultaneous deletion of *MIG1* and *MIG2* increases xylose consumption by 12% under constant fermentation conditions (Roca et al., 2004). Overexpression of a *MIG1* mutant further promotes xylose utilization in recombinant strains, though the underlying mechanism remains unclear (Xu et al., 2018). Additionally, co-deletion of *MIG1* and *SNF1* accelerates glucose metabolism but reduces xylose utilization (Cai et al., 2018). These findings suggest that the Snf1 regulatory network plays a multifaceted role in xylose metabolism, necessitating further research to unravel its precise regulatory mechanisms and optimize its modulation for efficient pentose sugar fermentation.

### Protein kinase A (PKA) pathway

PKA is a key regulator of growth and metabolism, driving cellular activities that support rapid carbon metabolism and proliferation while suppressing stress responses (Thevelein and de Winde, 1999). The PKA pathway regulates glucose-dependent growth through two major branches: *GPR1*/*GPA2* (extracellular glucose sensing) and **RAS*1/2* (intracellular glucose metabolism). Previous studies have shown that neither of these systems effectively responds to xylose, leading to impaired sugar co-fermentation (Brink et al., 2016). To circumvent this limitation, researchers have engineered constitutively active mutants (*GPA2^G132V^* and **RAS*2^G19V^*) and deleted *PDE1* and *PDE2*, which encode phosphodiesterases that increase PKA signaling and xylose utilization (Wu et al., 2020). Other studies have reported that the deletion of *IRA2*, which inhibits PKA activity via negative regulation of *RAS* or *BCY1* (a gene encoding a PKA inhibitor), increases the specific xylose consumption rates (Myers et al., 2019; Sato et al., 2016). A recent study showed that deleting *ISU1*, which is essential for Fe-S cluster assembly and critical for metabolic and respiratory functions, combined with *IRA2*, resulted in the highest xylose consumption and ethanol production rates. This effect was attributed to altered glucose signaling in *S. cerevisiae*, which enhanced xylose metabolism by modifying cellular sugar perception and response (Osiro et al., 2019). These findings suggest that modifying the cAMP/PKA pathway engineering could provide a broader approach to improving pentose co-fermentation.

## Enhancing Simultaneous Co-utilization of Mixed Sugars

Metabolic engineering has improved the ability of *S. cerevisiae* to utilize pentose sugars from pectin-rich biomass. However, efficient co-fermentation of glucose with other sugars remains challenging (Jansen et al., 2017; Tran et al., 2020).

### Adaptive evolution and 2-deoxyglucose selection for co-fermentation

2-Deoxyglucose (2DG), a structural analog of glucose, inhibits glycolysis in yeast and other cells (Soncini et al., 2020). Yeast cells can absorb 2DG but cannot metabolize it, triggering selective pressure that drives the evolution of resistance mechanisms (Lagunas and Moreno, 1992). Mutations in genes such as *HXK2*, *SNF1*, *GLC7*, and *REG1* disrupt 2DG's interference with glucose signaling, alleviating carbon catabolite repression (Soncini et al., 2020). In *S. cerevisiae*, 2DG resistance enabled glucose-maltose co-fermentation. Spontaneous 2-DG-resistant mutants from baker's yeast exhibited improved maltose uptake even in the presence of glucose, suggesting relief from glucose-induced repression. Results indicated that the 2-DG mutant strain DOG9 showed approximately 9-fold higher maltase activity and 12-fold higher invertase activity than the parental strain when cultivated in media containing both maltose and glucose (Rincón et al., 2001). Further analysis of the 2-DG-resistant strain MCD4 revealed upregulation of *MAL11* and *MAL31*, encoding maltose permeases, leading to enhanced maltose utilization alongside glucose (Orikasa et al., 2018).

Adaptive laboratory evolution using 2DG and xylose as carbon sources has revealed that yeast can evolve to co-ferment xylose and glucose while bypassing glucose repression (Lane et al., 2018). In one study, exposure to increasing 2DG concentrations yielded the 2DG-resistant mutant SR8#22, which co-ferment glucose and xylose simultaneously, albeit reduced rates. The wild-type xylose-fermenting strain SR8 consumed glucose at 1.74 ± 0.29 g/g/h and xylose at 0.31 ± 0.02 g/g/h sequentially, whereas SR8#22 consumed glucose at 0.23 ± 0.04 g/g/h and xylose at 0.19 ± 0.01 g/g/h simultaneously. Identified mutations in *GLK1* (265A > G), *HXK2* (1364ΔC), and *HXK1* (916T > C) contributed to this phenotype. Despite variations in mixed sugar consumption rates, this study demonstrates that reducing glucose phosphorylation alleviates glucose repression in *S. cerevisiae*. These findings indicate that intracellular glucose phosphorylation plays a crucial role in the metabolic regulation of mixed sugars.

### Simultaneous co-fermentation of cellobiose and xylose

Glucose repression limits the efficient fermentation of pectin-rich biomass hydrolysates by delaying the utilization of D-xylose, L-arabinose, and D-galacturonic acid. Cellobiose serves as an alternative glucose source that circumvents this repression, enabling co-fermentation. In engineered *S. cerevisiae*, intracellular hydrolysis of cellobiose via *N. crassa* transporters *CDT-1* and *CDT-2*, along with β-glucosidase *GH1-1*, prevents extracellular glucose accumulation and glucose-mediated inhibition of xylose metabolism (Galazka et al., 2010). The engineered strain DA24-16BT3 co-fermented cellobiose and xylose, achieving an ethanol yield of ~0.40 g ethanol/g sugar and a productivity of ~1 g/L/h (Ha et al., 2011a). This strategy not only facilitates simultaneous sugar utilization but also enhances fermentation efficiency compared to the sequential utilization of glucose and xylose. Beyond ethanol production, cellobiose fermentation pathways have also been shown to enhance the biosynthesis of bio-based chemicals. In mixed-sugar fermentations, glucose inhibits xylose uptake, requiring glucose-limited fed-batch fermentation for optimal yields (Zha et al., 2013). Replacing glucose with cellobiose allows continuous sugar consumption, increasing the productivity of xylitol and lactic acid (Oh et al., 2013; Turner et al., 2016). These modifications enhance fermentation efficiency and support a more sustainable process by diversifying carbon sources and reducing dependence on glucose. Co-fermentation strategies have been extended to other non-glucose sugars found in pectin-rich biomass, such as galactose. Similar to cellobiose-xylose co-fermentation, co-fermentation of cellobiose and galactose accelerates sugar consumption and improves fermentation efficiency. This approach broadens the applicability of mixed-sugar fermentation for biofuel and biochemical production (Ha et al., 2011b; Kim et al., 2012).

## Conclusion and Future Perspectives

Pectin-rich biomass represents an abundant yet underutilized resource with significant potential for sustainable biofuel and biochemical production. Advances in metabolic engineering have enabled *S. cerevisiae* to assimilate non-glucose sugars, such as D-galacturonic acid, L-arabinose, and D-xylose, by enhancing sugar transport, carbon flux, and redox balance. However, major challenges persist in achieving efficient and simultaneous co-fermentation of mixed sugars, overcoming glucose repression, and ensuring robust industrial performance. Addressing these metabolic constraints remains critical for realizing the full potential of pectin-rich biomass as a feedstock for biomanufacturing. Recent strategies in transporter engineering, adaptive laboratory evolution, and metabolic rewiring have improved sugar uptake and fermentation efficiency. Modulating intracellular redox balance has further optimized metabolic flux and sugar assimilation. Additionally, the engineering of regulatory networks, such as the Snf1 and PKA pathways, has alleviated glucose repression, facilitating co-fermentation of carbon sources derived from pectin-rich biomass. Despite these advances, further refinements are needed to enhance strain robustness, maximize substrate utilization, and achieve economically viable bioprocesses.

Scaling up these strategies for industrial application entails several practical challenges. Fermentation of pectin-rich hydrolysates often results in acetate accumulation, particularly during galacturonic acid metabolism, which diverts carbon flux and lowers product yield. In addition, the high NADPH demand for galacturonic acid reduction can disrupt redox homeostasis, especially in large-scale fermentations. Citrus waste hydrolysates present further complications due to D-limonene, a terpene that compromises membrane integrity and impairs yeast performance. Although steam stripping can remove limonene and improve fermentation efficiency, it increases process complexity and cost. Pectin-rich hydrolysates also contain diverse inhibitory compounds—such as weak acids, furans, phenolics, heavy metals, and pesticides—that can exert additive or synergistic toxicity on microbial metabolism. Moreover, variations in sugar composition and inhibitor concentrations, arising from differences in biomass types and pretreatment methods, hinder process standardization and scalability. Thus, industrial viability requires not only genetic optimization but also integrated process strategies to mitigate inhibitor toxicity and maintain redox balance at scale.

Future research should focus on expanding applications beyond bioethanol toward high-value biochemicals, including organic acids (e.g., itaconic acid, succinic acid), polyols, and platform chemicals. Comparative techno-economic assessments of different fermentation strategies are necessary to identify cost-effective approaches for industrial implementation. Developing integrated biorefinery models that combine enzymatic hydrolysis, fermentation, and downstream processing will be essential for optimizing resource utilization and minimizing waste. Additionally, developing efficient enzyme cocktails for pectin hydrolysis and scalable fermentation processes will accelerate the commercialization of pectin-rich biomass-based bioproduction. The future of pectin-rich biomass valorization requires a multidisciplinary approach that integrates metabolic engineering, process optimization, and system-wide biorefinery analysis. Innovations in synthetic biology, genome-scale metabolic engineering, and machine learning will enable more precise strain engineering and fermentation control. These advancements will contribute to the sustainable valorization of food waste, supporting circular bioeconomy initiatives and the development of next-generation biofuels and biochemicals.

## Figures and Tables

**Table 1.** Sugar profiles of pectin-rich residue hydrolysates

Feedstock	Pretreatment	Hydrolysis	Glc	GalUA	Ara	Gal	Xyl	Rha	Reference
Sugar beet pulp	Steam pretreatment at 5 bar pressure for 24 min	Enzymatic hydrolysis using cellulase 13L-C013L	25.9	14.4	23	6.2	1.7	2.4	Hamley-Bennett et al. (2016)
Apple pomace	Acid pretreatment, 72% sulfuric acid 16 min at 91°C	Viscozyme L and celluclast mixture for 100 h	13.1	20.6	10.9	3.1	-	-	Gama et al. (2015)
Mandarin peel	UV sterilization, freeze-dried	Polygalacturonase expressed by engineered, *Pichia pastoris*	39.4	27.3	3.3	3.9	2.4	2.9	Yang et al. (2020)
Orange peel	UV sterilization, freeze-dried	Polygalacturonase expressed by engineered, *Pichia pastoris*	35.5	25.6	5.6	2.7	2.2	2.1	Yang et al. (2020)
Jackfruit peel	Blanched, 80% (v/v) ethanol	2 M H_2_SO_4_ 110°C for 6 h	3.5	57.0	5.2	23.3	-	9.7	Li et al. (2019)
Melon peel	Microwave-assisted extraction with hexane and ethanol	Trifluoroacetic acid at 120°C for 75 min	14.4	40.8	30.1	8.3	-	1.2	Golbargi et al. (2021)
Passion fruit peel	Ultrasound-microwave-assisted three-phase partitioning	Trifluoroacetic acid at 110°C for 6 h	8.3	56.6	25.3	7.9	-	9.0	Zhao et al. (2023)
Watermelon rind	Freeze-dried, 96% (v/v) ethanol	2 M HCl in dry methanol for 5 h at 100°C	57.1	26.9	2.8	17.9	6.2	1.5	Mendez et al. (2021)

Content (%, w/w); g/100 g dry matter Parameters: Glc, glucose; GalUA, galacturonic acid; Ara, arabinose ; Gal, galactose; Xyl, xylose; Rha, rhamnose

**Table 2.** Reported sugar concentrations and bioproduct yields from pectin-rich residue hydrolysates using *S. cerevisiae*

Pectin-rich residue	Fermentation type	Hydrolysis	Fermentable sugars^1)^ (g/L)	pH	Ethanol yield (g/g)	Productivity (g/L/h)	Time (h)	Reference
Orange peel citrus waste	SSF	Pectinase, cellulase, β-glucosidase	5.93	6	0.67	1.50	24	Wilkins et al. (2007)
Orange peel citrus waste	SSF	Pectinase, cellulase, β-glucosidase	-	4.2–4.8	4.05% (w/v)	-	18	Zhou et al. (2008)
Mango peel hydrolysate	SHF	Pectinase, trizyme 50	150	5	0.476	0.99	72	Reddy et al. (2011)
Mandarin peel	SHF	In-house from enzyme HEA and HEB	68.5	4.8	0.43	3.28	9	Choi et al. (2015)
Grapefruit peel	SHF	In-house from enzyme HEA and HEB	51.8	4.8	0.42	2.40	9	Choi et al. (2015)
Lemon peel	SHF	In-house from enzyme HEA and HEB	46.8	4.8	0.42	2.18	9	Choi et al. (2015)
Lime peel	SHF	In-house from enzyme HEA and HEB	34.8	4.8	0.41	1.60	9	Choi et al. (2015)
Orange peel	CBP	Genome integration of the cellulase gene from *Ampullaria gigas* Spix	20.0	-	0.38	0.16	48	Yang et al. (2018)
Orange processing waste	SSF	Pectinase, cellulase	57.7	4.2	0.48	0.56	48	Widmer et al. (2010)
Sweet lime peel	SHF	Pectinase, cellulase	7.10	-	0.092	-	72	John et al. (2017)
Citrus peel waste	SHF	Acid hydrolysis	101	4.8	0.63	3.42	9	Kyriakou et al. (2020)
Orange citrus waste	SHF	Acid hydrolysis	29.0	5	0.43	1.65	24	Pourbafrani et al. (2010)

SSF, Simultaneous Saccharification and Fermentation; SHF, Separate Hydrolysis and Fermentation; CBP, Consolidated Bioprocessing; In-house enzyme from *Aspergillus citrisporus* (HEA) and *Trichoderma longibrachiatum* (HEB);

1) Sum of fermentable sugars (sucrose, glucose, fructose, and galactose)
